# 640. Efficacy of the Use of Bacteriophages to Combat *Pectobacterium atrosepticum*

**DOI:** 10.1093/ofid/ofad500.704

**Published:** 2023-11-27

**Authors:** Ian N Fabris, Melanie Kingett

**Affiliations:** Devon Preparatory School, Downingtown, Pennsylvania; Devon Preparatory School, Downingtown, Pennsylvania

## Abstract

**Background:**

This inquiry aimed to investigate the potential of bacteriophages vB_PatP_CB3, vB_PatP_CB4, and vB_PatP_CB5 as methods for controlling the spread of *Pectobacterium atrosepticum*, a bacterial pathogen that causes black-leg disease and extensive crop damage.

Structure of Podovirus

**Figure:**
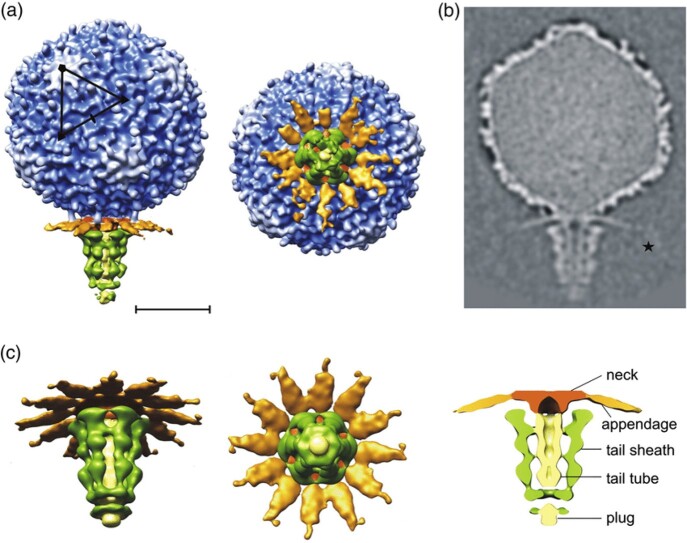

Morphology of typical N4-like podovirus (Anatomy of an N4-like Podovirus phage, (adapted from Insight into DNA and Protein Transport in Double-stranded DNA Viruses: The Structure of Bacteriophage N4, J. McPartland, et al.))

Soft Rot in Potato

**Figure:**
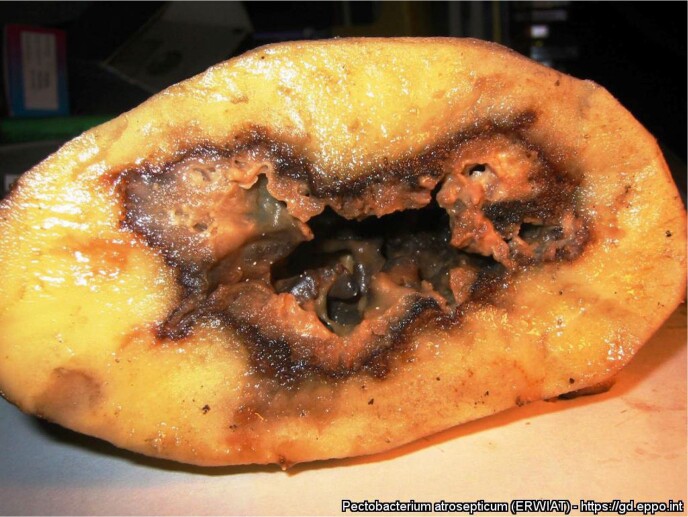

Pectobacterium atrosepticum infection in potato (Pectobacterium atrosepticum infection in Solanum tuberosum, the common potato (M. Kuznetsova All-Russian Institute of Phytopathology))

**Methods:**

Phages were initially selected based on the location of isolation of the phage and its proximity to a field with a known current, or recent previous, *Pectobacterium atrosepticum* infection among potato crops. Phages were narrowed down by the specificity of their receptor-binding proteins for the protein receptors on the bacterium cell wall, as determined by FASTA sequence comparison between phage tail fiber RBPs and bacterial integral glycoproteins. A standard serial dilution and phage titration protocol in conjunction with a plaque assay were subsequently used to evaluate the efficacy of each phage, both individually and in a cocktail.

Plaque Assay Diagram

**Figure:**
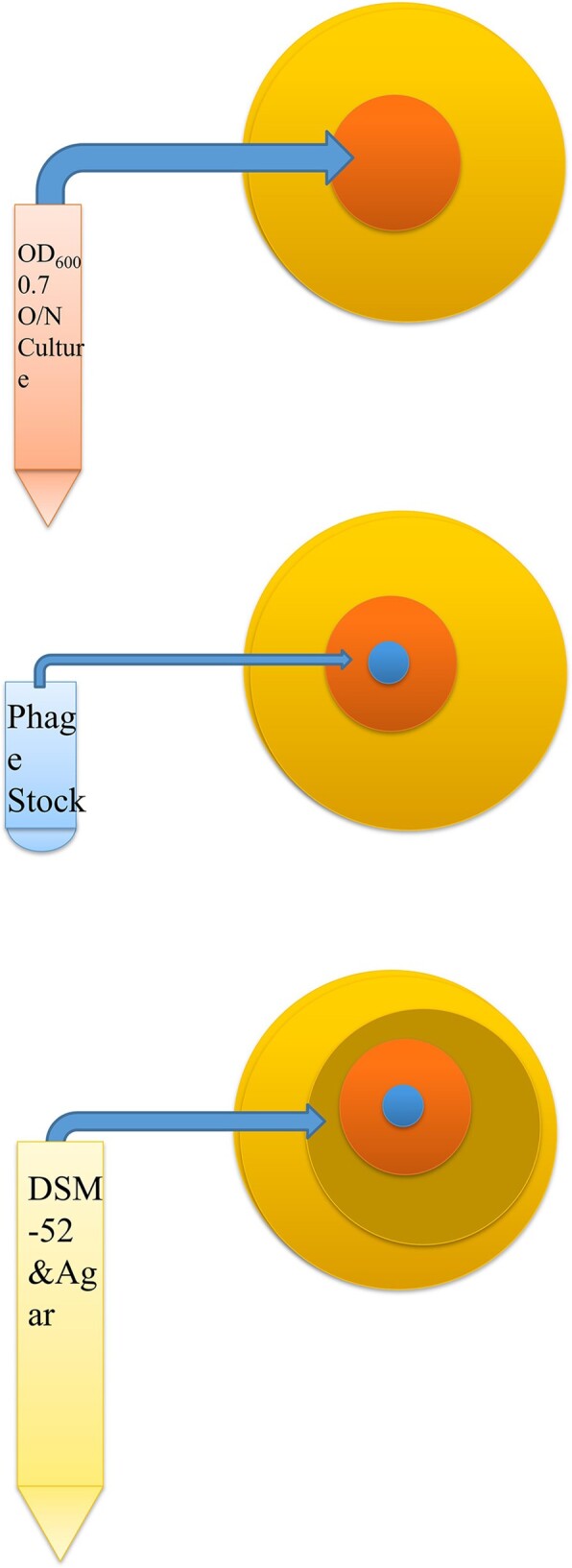

Standard plaque assay procedure used inoculate plates to obtain plaques

**Results:**

Plaque assays showed that vB_PatP_CB5 produced a large number of plaques, indicating successful cell lysis and bacteriophage propagation within the bacterial colony. In contrast, vB_PatP_CB3 and vB_PatP_CB4 produced no measurable plaques.

Plaques on Bacterial Lawn

**Figure:**
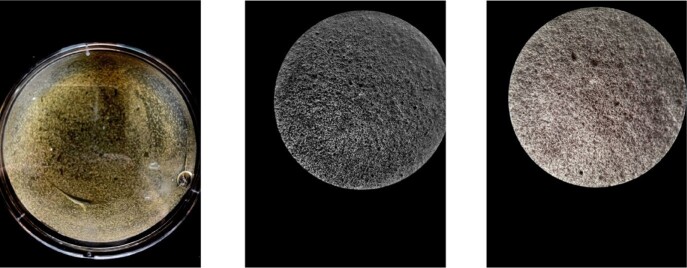

Observed plaque formation after initial 24 hour period

Plaque Charts from Trial 1

**Figure:**
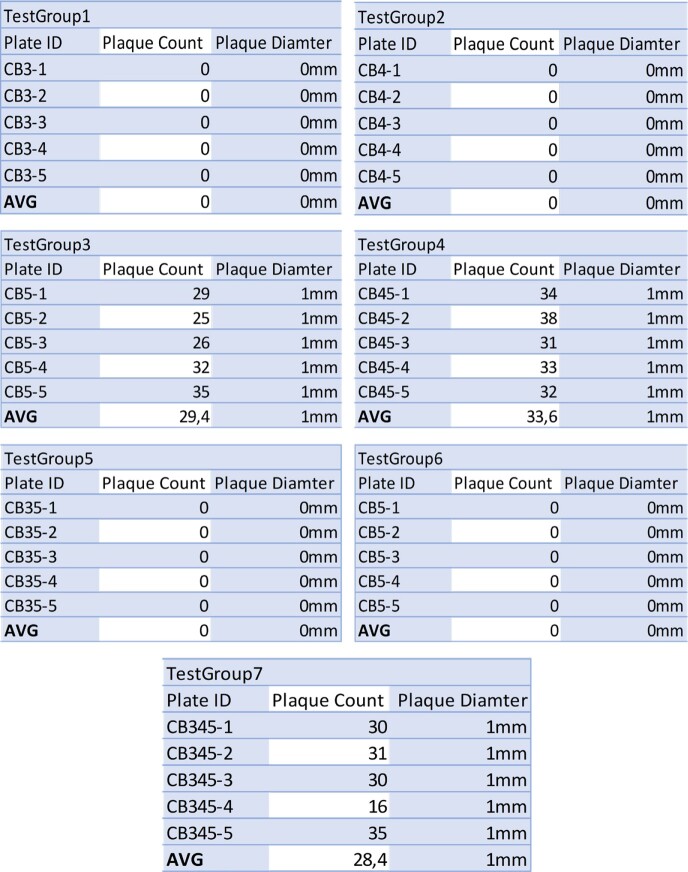

Plaque formation from trial 1

Plaque Charts from Trial 3

**Figure:**
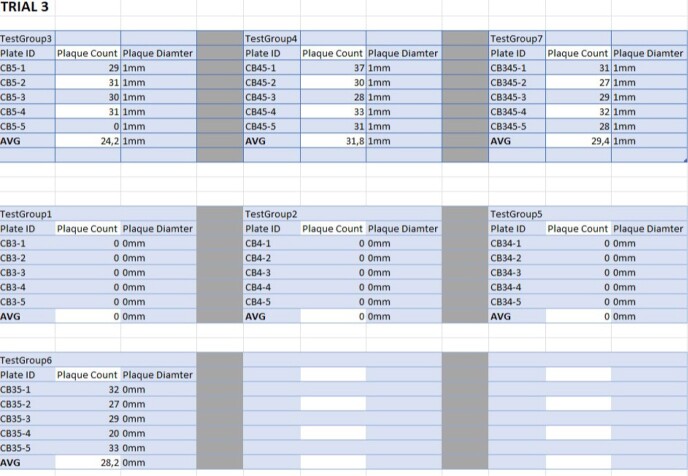

Plaque Charts from Trial 3

**Conclusion:**

Based on these findings, it was concluded that vB_PatP_CB5 is the most effective at lysing and controlling growth of *P. atrosepticum*. These results have implications for the development of new phage-based treatments for black-leg disease and for limiting the associated crop damage.

**Disclosures:**

**All Authors**: No reported disclosures

